# Discovery of a *hapE* Mutation That Causes Azole Resistance in *Aspergillus fumigatus* through Whole Genome Sequencing and Sexual Crossing

**DOI:** 10.1371/journal.pone.0050034

**Published:** 2012-11-30

**Authors:** Simone M. T. Camps, Bas E. Dutilh, Maiken C. Arendrup, Antonius J. M. M. Rijs, Eveline Snelders, Martijn A. Huynen, Paul E. Verweij, Willem J. G. Melchers

**Affiliations:** 1 Department of Medical Microbiology, Radboud University Nijmegen Medical Centre, Nijmegen, The Netherlands; 2 Nijmegen Institute for Infection, Inflammation and Immunity (N4i), Nijmegen, The Netherlands; 3 Centre for Molecular and Biomolecular Informatics, Nijmegen Centre for Molecular Life Sciences, Radboud University Nijmegen Medical Centre, Nijmegen, The Netherlands; 4 Unit of Mycology, Department of Microbiological Surveillance and Research, Statens Serum Institut, Copenhagen, Denmark; Imperial College Faculty of Medicine, United Kingdom

## Abstract

Azole compounds are the primary therapy for patients with diseases caused by *Aspergillus fumigatus*. However, prolonged treatment may cause resistance to develop, which is associated with treatment failure. The azole target *cyp51A* is a hotspot for mutations that confer phenotypic resistance, but in an increasing number of resistant isolates the underlying mechanism remains unknown. Here, we report the discovery of a novel resistance mechanism, caused by a mutation in the CCAAT-binding transcription factor complex subunit HapE. From one patient, four *A. fumigatus* isolates were serially collected. The last two isolates developed an azole resistant phenotype during prolonged azole therapy. Because the resistant isolates contained a wild type *cyp51A* gene and the isolates were isogenic, the complete genomes of the last susceptible isolate and the first resistant isolate (taken 17 weeks apart) were sequenced using Illumina technology to identify the resistance conferring mutation. By comparing the genome sequences to each other as well as to two *A. fumigatus* reference genomes, several potential non-synonymous mutations in protein-coding regions were identified, six of which could be confirmed by PCR and Sanger sequencing. Subsequent sexual crossing experiments showed that resistant progeny always contained a P88L substitution in HapE, while the presence of the other five mutations did not correlate with resistance in the progeny. Cloning the mutated *hapE* gene into the azole susceptible *akuB* ^KU80^ strain showed that the HapE P88L mutation by itself could confer the resistant phenotype. This is the first time that whole genome sequencing and sexual crossing strategies have been used to find the genetic basis of a trait of interest in *A. fumigatus*. The discovery may help understand alternate pathways for azole resistance in *A. fumigatus* with implications for the molecular diagnosis of resistance and drug discovery.

## Introduction

*Aspergillus fumigatus* is a ubiquitous saprophytic mold. Although humans inhale at least several hundred of *A. fumigatus* conidia per day, it rarely causes serious medical conditions in healthy individuals. In contrast, immunocompromised patients such as solid organ and hematopoietic stem cell transplant recipients and patients receiving chemotherapy are at risk of developing invasive aspergillosis, a commonly fatal infection [1]. Additionally, *A. fumigatus* is able to cause a wide range of other non-invasive diseases including allergic syndromes and aspergilloma, many of which require treatment with antifungal agents [1], [2].

Current treatment options of *Aspergillus* diseases include three classes of antifungal agents: polyenes (amphotericin B), echinocandins (caspofungin) and azoles, the latter being the largest and most widely used class of antifungal drugs [3], [4]. Voriconazole is the recommended first choice therapy for invasive aspergillosis [5], [6]. Itraconazole is commonly used for the treatment of chronic and allergic conditions [2] and posaconazole is effective in preventing invasive aspergillosis in patients with certain hematologic malignancies [7], [8].

Although *A. fumigatus* is generally susceptible to these azole antifungals, acquired resistance is increasingly being reported over the last few years [9]–[12]. Evidence is also accumulating that patients suffering from azole-resistant *Aspergillus* disease may fail to respond to therapy [11], [13]–[19]. The most common mechanisms of resistance in *A. fumigatus* are mutations in the *cyp51* gene encoding sterol 14α-demethylase, the target for azoles. Azoles inhibit this enzyme, thereby blocking its function in the ergosterol biosynthesis pathway, resulting in ergosterol depletion and accumulation of toxic sterols [20]. The *A. fumigatus* genome contains two distinct but closely related *cyp51* (*erg11*) genes: *cyp51A* and *cyp51B*, each encoding a different protein [21]. Mutations have rarely been detected in the *cyp51B* gene and have never been shown to be related to azole resistance [4]. In contrast, certain non-synonymous point mutations in the *cyp51A* gene (for example at codons G54, G138 and M220) correspond with azole resistance [22]–[24]. Other mutations responsible for azole resistance include tandem repeats of 34, 46 and 53 bp in the *cyp51A* promoter region (generally combined with mutations in the gene itself), resulting in an increased expression of the *cyp51A* gene [13], [25]–[27]. In addition, an *Aft1* transposon was found inserted 370 bp upstream of the start codon, possibly modulating *cyp51A* expression as well [28].

As is the case for *A. fumigatus*, azole resistance in other fungi such as *Candida* may be caused by alterations and over-expression of the azole target 14α-demethylase. Alternatively, over-expression of drug efflux transporters that pump the toxic azoles out of the cell have also been related to resistance in *Candida* [29], [30]. *A. fumigatus* contains several efflux pumps of the ATB-binding cassette (ABC) family and the Major Facilitator Superfamily (MFS) [31]. Although some of these transporters have been suggested to reduce azole susceptibility [31]–[35], none of them has been proven to play a direct role in resistance yet [28]. The question thus remains whether efflux transporters play an active role in azole resistance in *A. fumigatus* [34], [36]. An alternative azole resistance mechanism in *Candida* is a defective sterol Δ^5,6^-desaturase, encoded by the *erg3* gene. This allows the accumulation of less toxic sterols in the presence of azole antifungals [29], [31], [37]. However, the *erg3* genes have never been implicated in azole resistance in *A. fumigatus* [37].

Today, an increasing number of azole-resistant isolates are being reported without alterations in the *cyp51A* gene or promoter region [9]. However, no alternate resistance mutations have yet been found in clinical *A. fumigatus* isolates. Attempts to find new genes involved in azole resistance included determining expression levels of specific transporter genes [33]–[35], [38] and disruption, duplication, or truncation of a specific gene of interest followed by analysis of antifungal susceptibility in the recombinant [39]–[45]. Here, we apply a new strategy to unravel the resistance mechanism using a set of four sequentially isolated isogenic clinical *A. fumigatus* isolates that acquired an azole resistant phenotype during prolonged treatment. The isolates were obtained from a patient with chronic granulomatous disease (CGD) as described in detail in a previous study [46]. The first two isolates were susceptible to azole antifungals, but the last two isolates were azole resistant and the patient failed azole-echinocandin combination therapy. As the *A. fumigatus* isolates did not contain any mutations in the *cyp51A* gene or its promoter region, this set of isolates offered a unique opportunity to study the *de novo* acquisition of non-*cyp51A* azole resistance mutations. Our strategy included whole genome sequencing, sexual crossing experiments and gene replacements to identify the gene responsible for the observed resistance.

## Materials and Methods

### Origin and characterization of *A. fumigatus* isolates

**Figure 1:**
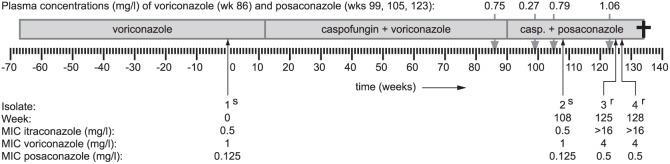
Relapsing infection with *A. fumigatus* in a CGD patient. During the course of the disease, four *A. fumigatus* isolates were obtained. Sampling dates of isolates 1 through 4 are indicated. S indicates susceptibility to azoles and R indicates resistance. Minimum inhibitory concentrations (MICs) for itraconazole, voriconazole, and posaconazole are also shown. The patient was treated with voriconazole monotherapy (week −67 to +12), caspofungin+voriconazole combination therapy (week +12 to +90) and caspofungin+posaconazole combination therapy (week +90 to +134). Plasma concentrations at week 86 (voriconazole) and weeks 99, 105, and 123 (posaconazole) are also indicated. The patient died from the pulmonary infection at week 134 [46].

The patient, origin of the isolates, and characterization of the isolates were described in detail in a previous publication [46]. The patient was a 21 year-old male with CGD. Due to a pulmonary infection caused by *A. fumigatus*, he was treated with multiple courses of antifungal therapy including caspofungin, voriconazole and a combination of the two. After relapse of the infection half a year later various treatment regimens were implemented including voriconazole, caspofungin/voriconazole combination therapy and caspofungin/posaconazole combination therapy. During this relapsing infection various respiratory samples were taken under routine care and four *A. fumigatus* isolates were obtained, designated isolates 1 through 4 (Figure 1). Unfortunately, despite antifungal treatment it was not possible to eliminate the fungus and the patient died from his pulmonary infection.

*In vitro* susceptibility testing of the patient isolates was performed using the EUCAST broth microdilution reference method [47]. Results were interpreted using the previously proposed breakpoints [12]. DNA was isolated and STR*Af* 3A, 3B, 3C, 4A, 4B, and 4C microsatellite genotypes were determined [48]. The *cyp51A* and *cyp51B* coding genes and promoter regions were amplified and sequenced as described before [48]. Sequences of the four patient isolates were aligned to identify any sequence differences in *cyp51A* and *cyp51B*.

### Complete genome sequencing and assembly

In order to unravel the resistance mechanism, isolates 2 and 3 (isolated 17 weeks apart, directly before and after the onset of azole resistance) were selected for complete genome sequencing (Figure 1). The isolates were cultured in liquid medium and after harvesting and drying mycelial mats, DNA was isolated as described elsewhere [49]. The Illumina Paired-End DNA sequencing Sample Prep Kit (cat #1001809) was used to process the DNA samples. Fragmentation of the DNA by nebulization, ligation of sequencing adapters and PCR amplification of the resulting product was performed according to the Illumina protocol Preparing Samples for Paired-end Sequencing (1005063 Rev. A). The quality and yield after sample preparation was measured with Lab-on-a-Chip analysis. The size of the resulting product was consistent with the expected size of approximately 300 bp after excision from an agarose gel. DNA sequencing using the Genome Analyzer II (Illumina) was performed according manufacturer's protocols. Two sequencing reads of 36 cycles each using the Read 1 sequencing and Read 2 sequencing primers were performed with the flow cell. Sequencing data are available at the Sequence Read Archive (SRA) at http://www.ebi.ac.uk/ena/data/view/ under accession number ERP001097. The two complete *A. fumigatus* genomes that are publically available (clinical isolates Af293 and A1163 [50], [51]) were used as reference sequences to aid the genome assembly. Assembly was done using Maq [52] with default parameters, taking into account the paired-end nature of the reads.

### Prioritization and validation of potential mutations

From the complete genome sequencing data, non-synonymous mutations were selected in isolate 3 (resistant) relative to isolate 2 (susceptible) and both reference isolates Af293 and A1163 (susceptible). Many of these mutations were expected to be unreliable because of low sequencing depth or low quality of the Maq consensus assembly. Potential mutations were prioritized based on the product of the Maq assembly quality scores in the susceptible and resistant assembly (further referred to as “product quality score”). As we did not know *a priori* what cutoffs to use, mutations with the highest product quality scores were selected for confirmation by conventional PCR and Sanger sequencing. Moreover, potential mutations with lower product quality scores were also validated for proteins potentially important in azole resistance (e.g. transporters), or if they occurred in the genomic vicinity of more reliably called mutations and could be validated in one sequencing experiment. For the selected mutations, primers were designed (Table 1) and used to amplify and sequence isolates 2 and 3 first. For confirmed mutations, isolates 1 and 4 were subsequently amplified and sequenced.

**Table 1:** Validation of potential mutations identified in the complete genome sequence of isolate 3 relative to isolate 2 as well as both reference strains.

Product quality score	Gene (abbreviated)	Gene ID in Af293/A1163	Primer ID	Primer sequence (5′-3′)	Result of validation
5670	*hypothetical*	AFUA_2G02180/AFUB_019270	411.33	GCATCACCAAAACACAGTGG	correct
			411.34	GGCAAATCTCCCAAGATTGA	
4320	*erg6*	AFUA_4G03630/AFUB_099400	411.19	GTATCGAGCTGGGTGATGGT	correct
			411.20	AATACATGGGCGTGAAGAGC	
3780	*hapE*	AFUA_6G05300/AFUB_092980	411.13	CCGCGACATACTAACGACCT	correct
			411.14	GCTGTCCCAGAGAACCGTAA	
3744	*erg25*	AFUA_4G04820/AFUB_098170	411.11	CCCAAAAAGGCAAGAAATGA	correct
			411.12	TCCGTTTGGACCATGATGTA	
3648	*alpha-amino*	AFUA_4G11240/AFUB_068270	411.35	CGGTTCTGAGGGTAGACCAA	correct
			411.36	GAAGCCCTCCTGGCTAGAGT	
3600	*C2H2 finger*	AFUA_5G07960/AFUB_055490	411.37	ACTTTGGGCTTTTGTTGTGG	correct
			411.38	GGAAGCAAGGTGGTTCTCAA	
2052	*origin recognition*	AFUA_8G04240/AFUB_083310	411.92	GGTCCCTTGTGTGAAGCAAT	incorrect
			411.93	GTCCCCCAGTCTTTTGGAAT	
2046	*hypothetical*	AFUA_5G09590/AFUB_057140	411.27	GTGGTTGATTGCAGGAGGTT	incorrect
			411.28	AAGCAGTCGATTCGCAGACT	
1701	*hypothetical*	AFUA_1G04630/AFUB_004980	411.29	CCCTGTCAGGTGATGTCCTT	incorrect
			411.30	AGGAACGTCATTGCGGATAC	
…..	*…..*	…..	…..	…..	…..
1596	*MRS7 family*	AFUA_3G08230/AFUB_040880	411.25	GCTTTTCTTGGCTGTTCGAG	incorrect
			411.26	GTGGACTCGCTCTCTGTTCC	
1008	*MRS7 family*	AFUA_3G08230/AFUB_040880	411.25	GCTTTTCTTGGCTGTTCGAG	incorrect
			411.26	GTGGACTCGCTCTCTGTTCC	
840	*MFS transporter*	AFUA_6G06680/AFUB_072610	411.15	AGTGCCGCTGAAGATCAGTT	incorrect
			411.16	ATGGACGGAAAACAGACGAG	
720	*hypothetical*	AFUA_1G04630/AFUB_004980	411.29	CCCTGTCAGGTGATGTCCTT	incorrect
			411.30	AGGAACGTCATTGCGGATAC	
348	*hypothetical*	AFUA_2G14620/AFUB_030250	411.23	GCAGTGGACTCATCCTCCTC	incorrect
			411.24	GTGAAGCAGTCCCTCCTCTG	
348	*hypothetical*	AFUA_1G04630/AFUB_004980	411.29	CCCTGTCAGGTGATGTCCTT	incorrect
			411.30	AGGAACGTCATTGCGGATAC	
69	*AtrD*	AFUA_4G08800/AFUB_065900	411.9	TACAAGGCCGAACAAAATCC	incorrect
			411.10	TCAGACGAGTGTTGGACCAG	
45	*MFS transporter*	AFUA_6G06680/AFUB_072610	411.15	AGTGCCGCTGAAGATCAGTT	incorrect
			411.16	ATGGACGGAAAACAGACGAG	
42	*RTA1 domain*	AFUA_5G01310/AFUB_049810	411.17	GACGCTGACCGTGTACAGAA	incorrect
			411.18	GTCTGCTCCTTCTGCTTGCT	

Potential mutations were prioritized using the product quality score (see Methods). Primers used for amplification and sequencing are indicated. The nine highest quality scores (above ‘…..’) as well as several mutations with lower scores (below ‘…..’) were validated. ‘Correct’ indicates that the mutation found by complete genome sequencing was validated using conventional PCR and sequencing methods; ‘incorrect’ indicates isolate 3 did not differ from isolate 2.

### Sexual reproduction and analysis of the progeny

The mating type of the isolates was determined as described before [53]. Isolates 1, 2, 3 and 4 were each crossed with isolate 47–169, a highly fertile environmental *A. fumigatus* isolate (kindly provided by C.M. O'Gorman and P.S. Dyer, School of Biology, University of Nottingham, Nottingham, UK). Mating experiments were performed on oatmeal agar plates (Difco Oatmeal agar) and inoculation was performed as described previously [54]. Plates were sealed with Parafilm and incubated at 30°C in the dark. Crosses were examined weekly and when cleistothecia developed, ascospore suspensions were obtained as described before [54]. The suspensions were heated at 70°C for one hour to prevent the germination of asexual spores [54]. Then small aliquots of the suspensions were plated onto Sabouraud agar plates and incubated for two days at 28°C. Germinating ascospores were transferred to Sabouraud agar slants and grown at 28°C for five days. The progeny were tested for their susceptibility to azoles as described above and DNA was isolated as described elsewhere [48]. The DNA was used to check for the presence or absence of the confirmed mutations by PCR and sequencing, using the same primers as used for validation (Table 1). In addition, from a selection of the progeny the microsatellite genotype was determined [48].

### Growth kinetic assay

Part of the progeny was selected to evaluate growth over time. For that, conidia were suspended in 0.1% Tween-20. Suspensions were adjusted to 1–4.2*10^6^ conidia/ml and diluted 1∶10 in water. 100 µl thereof was added to 100 µl of double strength RPMI 2% glucose medium in three individual wells of a microtiter plate. The plates were incubated at 37°C for 12 h inside a plate reader. The optical density (OD) at 450 nm was automatically recorded for each well every 30 minutes without shaking. For each time point the OD of the triplicate was averaged and the changes in OD over time were used to generate growth curves. The maximum growth per hour during the exponential section of the curve was averaged for the susceptible and resistant progeny and to test whether differences where significant, student's t-test was applied (one-tailed, heteroscedastic).

### RNA isolation and *cyp51A* expression

A selection of the progeny and parental isolates were subjected to *cyp51A* expression analysis. The isolates were cultured twice and used for separate total RNA isolation. cDNA amplification and RT-PCR for *cyp51A* and *actin* expression levels were performed as described before [46]. The change in gene expression was determined using the *cyp51A/actin* ratio.

### Transformations

Gene replacement experiments were performed as described before [22], [26]. Briefly, DNA fragments of the six genes with mutations (Table 2) were obtained by PCR amplification using genomic DNA of the first resistant isolate 3. As a positive control, a fragment with a mutation in *cyp51A* known to be correlated with azole resistance (the *cyp51A* G54E substitution in clinical isolate AF-72 [22]), was amplified. The primers used to amplify the six genes as well as the *cyp51A* gene for the positive control are shown in Table 2. All primers were designed to include the complete gene, except for alpha-aminoadipate reductase large subunit (reference Af293 gene AFUA_4G11240 and A1163 gene AFUB_068270). Because of the large size of this gene, primers were designed approximately 1000 bp on both sides of the mutation. The PCR products were used for homologous gene replacement by electroporation into the azole susceptible isolate *akuB* ^KU80^ [26], [55]. Transformation mixtures were spread-plated on minimal-medium plates containing itraconazole (0.5 to 4 mg/l) to select for resistant recombinants. After three days of incubation at 37°C colonies were subcultured for further investigation. DNA was isolated and the recombinants were analyzed for the presence of the mutation by using the corresponding primers listed in Table 1. In successful recombinants, the complete mutant gene (primers Table 2) and the *cyp51A* gene and promoter region (as described above) were sequenced to ensure no other mutations were introduced. The susceptibility profiles of the successful recombinants (two recombinants were selected for each mutation) and the transformation recipient isolate *akuB* ^KU80^ were determined as described above.

**Table 2:** Primers used to amplify the six validated genes as well as *cyp51A* (positive control).

Gene (abbreviated)	Gene ID reference isolates Af293/A1163	Primer ID	Forward/reverse primer	Primer sequence (5′-3′)
*hypothetical*	AFUA_2G02180/AFUB_019270	411.55	forward	CTTTTGACATTTCCATATCAGTCCT
		411.56	reverse	TGACTATCTGTCACAATGGTTGTTC
*erg6*	AFUA_4G03630/AFUB_099400	411.39	forward	TTTCAAGATTGTGATCTTGTGGATA
		411.40	reverse	GACAGCCACGTCTATAAGTTTAGGA
*hapE*	AFUA_6G05300/AFUB_092980	411.47	forward	CTCTCGCGATACATTACTGTTGTTT
		411.48	reverse	AGTATACTCGTTAACCTGCCAATGA
*erg25*	AFUA_4G04820/AFUB_098170	411.43	forward	TTCAACACCCAGTTGTCTCATACTA
		411.44	reverse	CTGTCATCGATTTATGATAGCAGTG
*alpha-amino*	AFUA_4G11240/AFUB_068270	411.57	forward	GTATGTGGTCCTGTCGTTCATTC
		411.58	reverse	GGCAGAATCATCATCCTTGAG
*C2H2 finger*	AFUA_5G07960/AFUB_055490	411.53	forward	CGGTACCTTTACTGATCACTTTGAA
		411.54	reverse	GCTTCGTTTCACTCAATTCTATTTC
*cyp51A*	AFUA_4G06890/AFUB_063960	365.1	forward	ATGGTGCCGATGCTATGG
		365.2	reverse	CTGTCTCACTTGGATGTG

### *HapE* sequencing in resistant isolates without *cyp51A* mutations

Our fungal culture collection contains *A. fumigatus* clinical isolates from patients admitted to our hospital, as well as environmental isolates and isolates sent to our laboratory for identification, *in vitro* susceptibility testing, or research purposes. Only accredited scientists in our laboratory have access to the fungal collection. The collection was searched for isolates resistant to one or more azoles but without any *cyp51A* gene mutations known to be involved in resistance. Of these isolates *hapE* was amplified by PCR and subsequently sequenced (primers Table 2). Gene sequences were compared with isolate 2 to detect any mutations.

## Results

### Characterization of the isolates

The azole minimum inhibitory concentrations (MICs) of the four isolates obtained during the course of the disease in a CGD patient are shown in Figure 1. According to the suggested breakpoints [12], isolates 1 and 2 were azole susceptible, while isolate 3 and 4 were resistant to both itraconazole and voriconazole and had an intermediate susceptibility to posaconazole. The isolates did not have any nucleotide differences in their *cyp51A* and *cyp51B* genes and promoter regions (results not shown) and microsatellite genotyping showed that the four isolates had identical genotypes and were thus isogenic (results not shown).

### Genome sequencing, assembly and validation of potential mutations

As no *cyp51A* and *cyp51B* mutations developed, a new, unknown resistance mutation must have emerged during therapy. Due to the isogenic nature of the isolates, we anticipated that the most straightforward approach to identify this resistance mechanism would be by complete genome sequencing of isolates 2 and 3 (isolated directly before and after the onset of azole resistance). The volume of sequence data obtained using Illumina technology included 8,611,525 36-nt read pairs for isolate 2 and 4,808,633 36-nt read pairs for isolate 3. The total sequence volume was 620,029,800 nt (isolate 2) and 346,221,576 nt (isolate 3). After reference-guided assembly, the sequence coverage of isolate 2 was 93.7% (mapped against Af293) and 94.9% (mapped against Af1163). For isolate 3 this was 93.6% (mapped against Af293) and 94.8% (mapped against Af1163). The average sequencing depth was 20.3 reads for isolate 2 and 11.5 for isolate 3. Sixty-nine potential mutations were identified in resistant isolate 3 that were non-synonymous relative to both the susceptible isolate 2 and both reference isolates Af293/A1163 (Table S1). The potential mutations were then prioritized based on their product quality scores. Mutations with the highest scores as well as a selection of genes with lower scores were validated by PCR and Sanger sequencing. Table 1 shows that only the six mutations with the highest product quality scores could be confirmed, the other mutations were false-positive calls. Subsequent testing of isolate 1 and 4 confirmed the presence of these six non-synonymous mutations in both resistant isolates 3 and 4 relative to the susceptible isolates 1 and 2 (Table 3).

**Table 3:** The six confirmed non-synonymous mutations, together with results of complete genome sequencing and validation.

Gene function	Protein ID Af293/A1163	Nucleotide in complete genome sequence isolate 2/3	Validated nucleotide in isolate 1/2/3/4	Amino acid substitution
Conserved hypothetical protein	AFUA_2G02180/AFUB_019270	C/T	C/C/T/T	S219L
Sterol 24-c-methyltransferase, putative (Erg6)	AFUA_4G03630/AFUB_099400	T/G	T/T/G/G	W320G
CCAAT-binding factor complex subunit HapE	AFUA_6G05300/AFUB_092980	C/T	C/C/T/T	P88L
C-4 methyl sterol oxidase, putative (Erg25)	AFUA_4G04820/AFUB_098170	G/A	G/G/A/A	W218*
Alpha-aminoadipate reductase large subunit, putative	AFUA_4G11240/AFUB_068270	T/C	T/T/C/C	F481S
C2H2 finger and ankyrin domain protein, putative	AFUA_5G07960/AFUB_055490	T/A	T/T/A/A	Y347*

* refers to a premature stop codon.

### Sexual reproduction and analysis of the progeny

To elucidate which of the six mutations was the cause of resistance, sexual crosses were performed. Note that this approach would also identify potential combinations of mutations if two or more mutations were together responsible for the resistant phenotype. Isolates 1 to 4 were of mating type MAT1-2 and were crossed with 47–169, an isolate of the opposite mating type MAT1-1. After six weeks of incubation, cleistothecia appeared on the agar plate. Ascospore progeny were isolated and subcultured. Ten (for isolates 1 and 2), forty (for isolate 3) and twenty (for isolate 4) progeny isolates were randomly selected for susceptibility testing and azole resistant isolates were present in the progeny of isolates 3 and 4, but not in the progeny of isolates 1 and 2 (Table 4). The resistant progeny all exhibited a MIC of >16 mg/l for itraconazole. In addition, these isolates showed elevated MICs for voriconazole and posaconazole, similar to those observed in patient isolates 3 and 4 (results not shown). In addition, a minority of the progeny of the resistant isolates 3 and 4 exhibited an altered susceptibility phenotype for itraconazole, referred to as trailing effect [56].

**Table 4:** Distribution of susceptible and resistant isolates in the sexual crossing progeny of isolate 47–169 and each of the four isolates in this study.

Phenotype	Progeny isolate 1	Progeny isolate 2	Progeny isolate 3	Progeny isolate 4
Susceptible	10 (100%)	10 (100%)	25 (63%)	11 (55%)
Trailing-endpoint itraconazole	-	-	8 (20%)	3 (15%)
Resistant	-	-	7 (18%)	6 (30%)
Total	10 (100%)	10 (100%)	40 (100%)	20 (100%)

The forty progeny of isolate 3 were screened for the presence of the six mutations validated in the whole genome sequencing analysis. All mutations were present in the susceptible as well as in the resistant progeny, except for the mutation in the *hapE* gene which was only present in resistant isolates. Furthermore, Table 5 shows that all resistant progeny of isolate 3 and 4 contained the *hapE* mutation while the other mutations were only present in a part of the resistant progeny. Microsatellite typing (Table 6) showed that twelve out of the thirteen resistant progeny (92%) had a unique microsatellite genotype, indicating that unique germinating ascospores harboring the resistance mutation were isolated and not a single clone of resistant progeny. Taken together, these results strongly suggest that the mutation in *hapE* caused the observed resistant phenotype.

**Table 5:** Resistant progeny of crosses 47–169 x isolate 3 and 47–169 x isolate 4: results of susceptibility testing and mutation screening.

Isolate		MIC (mg/l)			Gene^a^					
		ITZ	VOR	POS	hypoth.	*erg6*	*hapE*	*erg25*	*alpha.*	*C2H2*
47–169	parent	0.25	0.5	0.063	−	−	−	−	−	−
isolate 3	parent	>16	4	0.5	+	+	+	+	+	+
isolate 4	parent	>16	4	0.5	+	+	+	+	+	+
v121-42	progeny (47–169 x isolate 3)	>16	2	0.5	−	+	+	−	−	+
v121-43	progeny (47–169 x isolate 3)	>16	2	0.5	−	+	+	+	−	−
v121-54	Progeny (47–169 x isolate 3)	>16	2	0.5	+	+	+	+	−	−
v121-55	progeny (47–169 x isolate 3)	>16	2	0.5	−	+	+	−	−	−
v121-59	progeny (47–169 x isolate 3)	>16	2	0.5	−	+	+	−	−	−
v121-74	progeny (47–169 x isolate 3)	>16	4	0.5	−	+	+	−	+	+
v121-78	progeny (47–169 x isolate 3)	>16	2	0.5	−	+	+	−	+	−
v122-02	progeny (47–169 x isolate 4)	>16	2	0.5	−	+	+	+	+	−
v122-08	progeny (47–169 x isolate 4)	>16	4	0.5	−	+	+	+	+	−
v122-12	progeny (47–169 x isolate 4)	>16	2	0.25	−	−	+	−	−	−
v122-13	progeny (47–169 x isolate 4)	>16	4	0.5	−	+	+	+	+	−
v122-17	progeny (47–169 x isolate 4)	>16	2	0.25	−	−	+	−	+	−
v122-18	progeny (47–169 x isolate 4)	>16	2	0.5	−	−	+	+	−	−

a ‘−’ indicates absence of the mutation, ‘+’ indicates presence of the mutation.

**Table 6:** Microsatellite typing results of the resistant progeny.

Isolate		Microsatellite						Microsatellite genotype
		3A	3B	3C	4A	4B	4C	
47–169	parent	16	10	24	11	14	9	A
isolate 3	parent	47	13	13	7	9	10	B
isolate 4	parent	47	13	13	7	9	10	B
v121-42	progeny (47–169 x isolate 3)	16	13	24	7	9	9	C
v121-43	progeny (47–169 x isolate 3)	16	13	13	7	9	10	D
v121-54	progeny (47–169 x isolate 3)	16	13	13	7	14	10	E
v121-55	progeny (47–169 x isolate 3)	16	13	24	7	9	10	F
v121-59	progeny (47–169 x isolate 3)	16	13	13	11	14	10	G
v121-74	progeny (47–169 x isolate 3)	16	13	13	7	9	9	H
v121-78	progeny (47–169 x isolate 3)	16	13	24	7	14	10	I
v122-02	progeny (47–169 x isolate 4)	16	13	24	7	9	9	C
v122-08	progeny (47–169 x isolate 4)	47	13	13	7	14	9	J
v122-12	progeny (47–169 x isolate 4)	47	13	13	7	14	10	K
v122-13	progeny (47–169 x isolate 4)	47	13	24	7	9	10	L
v122-17	progeny (47–169 x isolate 4)	47	10	24	7	9	9	M
v122-18	progeny (47–169 x isolate 4)	47	13	13	7	9	9	N

**Figure 2:**
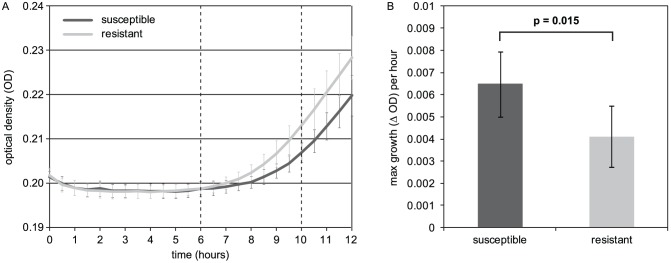
*In vitro* growth curves of susceptible and resistant progeny. A. *In vitro* growth curves of susceptible (n = 12) and resistant (n = 4) progeny. B. The maximum growth per hour during the exponential phase of the growth curve (6–10 hours, between dashed lines of Figure 2A) was averaged (±standard deviation) for susceptible and resistant progeny. The difference is significant according to a Student's T-test (one-tailed, heteroscedastic).

**Figure 3:**
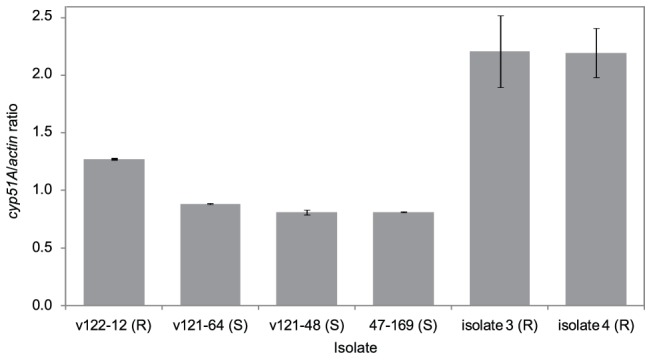
*Cyp51A* mRNA levels in the parental isolates (47–169, isolate 3, isolate 4) and three of the progeny (v122-12, v121-64, v121-48). Isolate v122-12 contains only the HapE substitution and not the other five gene mutations. As a control, we selected two other progeny: v121-64 containing only the *erg25* mutation and v121-48 containing the *erg6/erg25* double mutation. S: susceptible, R: resistant.

Sixteen of the progeny as well as both parental isolates (isolate 3 and 47–169) were selected to determine *in vitro* growth curves (Figure S1). As can be observed in Figure 2, growth rate for susceptible progeny was significantly higher compared to the resistant progeny (p = 0.015). In order to determine if the *cyp51A* expression level of progeny containing the HapE substitution was altered, RT-PCR experiments were performed. A ∼1.5-fold induction of the transcriptional profile of the *hapE*-containing progeny isolate compared to two susceptible progeny isolates was found (Figure 3).

### Transformations

Homologous gene replacement studies with the six mutated genes from isolate 3 were performed using *cyp51A* G54E as a positive control (Table 2). After selection on itraconazole-containing medium, successful recombinants were only obtained from the positive control *cyp51A* and from candidate resistance gene *hapE* (two recombinants each, Table 7). Subsequent sequencing of the complete *cyp51A* gene and promoter region and the *hapE* gene showed no additional mutations, indicating that only the P88L- (HapE) or the G54E- (Cyp51A) causing mutation was introduced by the homologous gene replacement. The positive control substitution G54E in Cyp51A is known to cause resistance to itraconazole and posaconazole [22], [33], [57]–[58], and indeed both recombinants showed resistance to itraconazole and posaconazole. Moreover, both HapE P88L recombinants showed resistance to itraconazole with an MIC of >16 mg/l, as was also observed in patient isolates 3 and 4 (cf. Figure 1 and Table 7). As observed in the patient isolates, the voriconazole and posaconazole MICs increased two dilution steps from 0.25 to 1 and from 0.032 to 0.125, respectively (cf. isolates 2 and 3 in Figure 1). These results show that the HapE P88L substitution is the only one of the six mutations in isolate 3 capable of conferring the azole resistant phenotype.

**Table 7:** MICs of recombinants with either the G54E substitution in Cyp51A (positive control) or the P88L substitution in HapE.

	Substitutions		MIC		
Isolate	Cyp51A	HapE	ITZ	VOR	POS
Transformation recipient *akuB* ^KU80^	-	-	0.125	0.25	0.032
Positive control Cyp51A (1)	G54E	-	>16	0.125	0.5
Positive control Cyp51A (2)	G54E	-	>16	0.125	1
HapE (1)	-	P88L	>16	1	0.125
HapE (2)	-	P88L	>16	1	0.125

MIC, minimum inhibitory concentration; ITZ, itraconazole; VOR, voriconazole; POS, posaconazole.

### *HapE* sequencing in resistant isolates without *cyp51A* mutations

Our collection contained eleven resistant *A. fumigatus* isolates without *cyp51A* mutations known to cause resistance. Eight were clinical isolates and the remaining three isolates were of environmental origin. Although some of the isolates did show polymorphisms in intronic regions of the gene, we never observed the mutation resulting in P88L or any other mutation within the exonic regions.

## Discussion

In our study the whole genome sequencing strategy was used to identify a resistance mutation that arose as a *de novo* mutation in a CGD patient with chronic fibrotic pulmonary aspergillosis caused by *A. fumigatus*. We selected two *A. fumigatus* isolates, taken just before and just after the transition from a susceptible to an azole-resistant phenotype. Although the resistant strain (isolate 3) was recovered only 17 weeks after the susceptible strain (isolate 2), we identified no less than six non-synonymous mutations. Moreover, if we use the validated product quality score as a reliable cutoff (3,000), we found 16 additional mutations, bringing the total number of mutations to at least 22 (Table S2). These included five mutations in non-coding regions and 11 cases where the resistant strain was synonymous to the susceptible reference genomes *A. fumigatus* Af293 and *A. fumigatus* A1163 and thus unlikely to be responsible for the resistant phenotype. This may indicate that *A. fumigatus* undergoes many genetic changes during human infection, possibly as a response to the stressful environment. Potentially, the resistant strain diverged from the dominant susceptible lineage even before the last susceptible strain was isolated. This minority strain may have been accumulating neutral or slightly deleterious mutations before finally the resistance causing mutation allowed it to dominate the *A. fumigatus* population in the CGD patient.

Because we identified more than one non-synonymous mutation, we chose sexual crossing as an additional selection method. After crossing the patient isolates with an azole susceptible isolate that did not contain any of the six mutations, the mutation in *hapE* was identified as the most likely cause of azole resistance as this was the only mutation present in all resistant progeny. Subsequent gene replacement experiments provided the final proof that the HapE P88L substitution was the cause of the resistance phenotype observed.

With respect to the sexual reproduction experiment, part of the progeny of isolates 3 and 4 exhibited trailing growth (partial inhibition of growth over an extended range of antifungal concentrations [59]) in the presence of itraconazole. However, these trailing isolates did show clear susceptible MIC endpoints for voriconazole and posaconazole. Trailing endpoints caused problems when testing *Candida* spp. with azoles as well, but animal models and clinical experience argue that trailing endpoints do not indicate true resistance [56], [60]–[61], suggesting that the progeny with trailing endpoints for itraconazole might not be truly resistant.

Furthermore, we noted that after sexual reproduction, only 18–30% of the progeny contained the *hapE* mutation instead of the expected 50%. Using *in vitro* growth kinetics in fluid medium, it was previously shown that the resistant isolates 3 and 4 have a significantly reduced growth rate and especially delayed germination compared to susceptible isolates 1 and 2, possibly associated with the resistance mechanism [46]. Therefore we hypothesized that the lower than expected recovery rate of the *hapE* mutation in the progeny might be due to slow germination, as we isolated the germinated sexual spores based on their growth on the medium and not by randomly selecting ascospores by using the microscope. Possibly only the faster germinating spores were collected resulting in a lower percentage of the slower germinating, resistant (*hapE* mutated) progeny. Determination of the *in vitro* growth curves confirmed a significant decrease in growth rate of azole resistant compared to azole susceptible progeny (Figure 2 and Figure S1). Furthermore, we could speculate that the resistance mechanism is indeed associated with loss of fitness (alteration of growth and virulence) observed in the resistant isolates, as suggested before [46]. We presume that the resistant isolates described here are disadvantaged in the absence of azoles as a consequence of the *hapE* mutation. In contrast, for isolates with mutations in *cyp51A*, there is no evidence of fitness loss *in vivo* [Mavridou E, Meletiadis J, Arendrup MC, Melchers WJ, Mouton JW, Verweij PE. Impact of CYP51A mutations associated with azole-resistance on *in vitro* growth rates and in vivo virulence of clinical *Aspergillus fumigatus* isolates. 20**^th^** European Congress of Clinical Microbiology and Infectious Diseases (ECCMID), Vienna, April 2010; abstract O345].

Remarkably, two of the five other validated mutations were situated in genes involved in the ergosterol biosynthesis pathway (*i.e. erg6* and *erg25*). *A. fumigatus* contains two potentially redundant copies of these genes (*erg6*: AFUA_4G03630/AFUB_099400 and AFUA_4G09190/AFUB_066290; *erg25*: AFUA_4G04820/AFUB_098170 and AFUA_8G02440/AFUB_084150; as determined by the CADRE genome browser [62], [63] and literature [64], [65]). Both genes function downstream of the *cyp51* (*erg11*) gene, at least in *Saccharomyces cerevisiae* [66]. It has been shown that downstream genes *erg3* [29], [31], [37], [67], *erg6*, and *erg28* [67] are involved in resistance in other fungi. In addition, *erg6* and *erg25* are both up-regulated in amphotericin B/fluconazole resistant *C. albicans*, suggesting a possible role of these two genes in acquired resistance [68]. Neither *erg6* nor *erg25* have ever been described to impact on azole susceptibility in *A. fumigatus*, but many *erg* genes, amongst which *erg25*, have been shown to be differentially expressed after voriconazole exposure [69]. Nevertheless, we found no evidence that the mutations in either *erg6* or *erg25* are associated with the resistant phenotype in isolates 3 and 4; neither from the sexual crossing- nor from the transformation experiments. In addition, the *erg6*/*erg25* double mutation was found in azole susceptible progeny, excluding the possibility that both mutations together would result in the resistant phenotype (results not shown).

The *hapE* gene encodes the CCAAT-binding factor complex subunit HapE. Besides HapE, the CCAAT-binding complex (Hap-complex) consists of at least two other subunits: HapB and HapC. The complex is a transcription factor that specifically recognizes the regulatory CCAAT element found in the forward or reverse orientation in promoter regions of numerous eukaryotic genes [70]. The *hapE* gene contains four exons and the mutation is situated in the fourth exon, in the evolutionary conserved core domain [62], [63], [71], suggesting that P88 is an important amino acid for the function of the protein. Moreover, in the human NF-YC (the mammalian HapE homologue) deletion of residues 43–45 (homologous to *A. fumigatus* residues 87–89), are deleterious for DNA binding [72], indicating that P88 is needed for the Hap-complex to bind the regulatory CCAAT element and initiate transcription.

Initially, we hypothesized that the resistance observed in the HapE-mutant might be HapX-mediated. HapX has been shown to physically interact with the Hap-complex (as shown in *A. nidulans* [73]) and is important in adaptation to conditions of iron starvation. Conversely, in conditions of iron sufficiency, HapX expression is repressed [65]. HapX deficiency causes significant attenuation of virulence in a murine model of aspergillosis [65]. As our resistant patient isolates also showed reduced virulence [46], we tested the isolates for their azole susceptibility in the presence and absence of iron to investigate whether the resistance (and attenuated virulence) observed could be HapX-mediated. However, our results indicated that the presence of iron did not have any effect on susceptibility (results not shown), suggesting that resistance may not be mediated by HapX.

We have previously observed that the *cyp51A* expression level of isolates 3 and 4 was four to six-fold higher compared to isolates 1 and 2 [46]. In addition, analysis of the sexual progeny showed that the *hapE* mutant has an increased *cyp51A* mRNA expression compared to progeny without the *hapE* mutation. As Hap is a transcription factor complex, the increased resistance might be due to a gain of function mutation if the mutated Hap-complex binds to a CCAAT-box in the promoter region of *cyp51A* and induces the expression of the gene. Alternatively, Hap might function as a transcriptional repressor, and the HapE P88L substitution could de-repress *cyp51A* expression. A CCAAT-box is present in the 5′ upstream region of *A. fumigatus cyp51A*, located at −197 bp from the start codon, though additional analyses are needed to confirm the binding of the (mutated) Hap-complex. Gain-of-function mutations in transcription factors have already been shown to be linked to increased antifungal drug resistance in *C. albicans* [74]–[79].

The NF-Y (the mammalian Hap-complex)/SREBP (sterol-regulatory element binding protein) combination regulates essentially all genes involved in cholesterol metabolism [80], indicating that the fungal ergosterol biosynthesis (the pathway analogous to the mammalian cholesterol biosynthesis), may also be mediated by these regulators. An *in vitro* null-mutant of the *A. fumigatus* SREBP transcription factor SrbA has recently been shown to be highly susceptible to fluconazole and voriconazole [45]. A subsequent study suggested that the increased fluconazole susceptibility in the absence of SrbA was partially due to loss of iron homeostasis [81]. Furthermore, SrbA is a direct transcriptional regulator of *cyp51A*, being the likely underlying mechanism of the increased susceptibility of the deletion mutant [82]. It currently remains unclear whether the declined expression is indeed the cause of the increased azole susceptibility and there has been no evidence for involvement of SrbA in resistance in clinical cases. However, these data indicate that alterations in SREBP transcription factors such as SrbA or NF-Y (Hap) transcription factor subunits such as HapE may indeed initiate changes altering *cyp51A* expression and subsequently lead to azole resistance.

In conclusion, by combining a comparative genomic and genetic approach, we have discovered a novel triazole resistance mutation in *A. fumigatus* caused by a single base substitution in the transcription factor subunit HapE. We were fortunate to have a set of isogenic strains, *i.e.* isolates with a similar genetic background, and a comparison of the whole genome sequences of isolates 2 and 3 shortlisted 69 potential non-synonymous mutations. Through resequencing of candidate mutations, and the addition of sexual crosses, we were able to significantly reduce the selection of candidate mutations for the azole resistant phenotype.

Over the last decade, an increase of azole resistance in *A. fumigatus* isolates has been observed [9]–[12]. Importantly, patients suffering from azole-resistant *Aspergillus* disease may fail to respond to therapy [11], [13]–[19]. Insight in the mutations that cause resistance can help to understand the epidemiology, find new targets for antifungal compounds and to develop diagnostic tools. The need to identify resistance mechanisms was underscored in a recent paper estimating that in 54% of patients with an azole resistant *A. fumigatus* isolate the resistance mechanism was not Cyp51A-mediated and thus remained unexplained [9]. This is a challenge for the early diagnosis of azole-resistance in patients with *Aspergillus* diseases. In these patients fungal cultures often remain negative, which precludes *in vitro* susceptibility testing. Furthermore, a recent study showed that the use of molecular tools for detection of *cyp51A*-mutations directly in clinical specimens was much more sensitive than culture [83]. However, knowledge of underlying resistance mechanisms is required in order to apply molecular techniques in patient care. The strategy that we have described will help to identify resistance mechanisms and improve our diagnostic tools in order to ultimately improve the outcome of patients with azole resistant *Aspergillus* diseases.

## Supporting Information

Figure S1 ***In vitro*** **growth curves of parental isolates (isolate 3 and 47–169) as well as sixteen of their progeny. S: susceptible, R: resistant.**
(XLS)

Table S1 Potential non-synonymous mutations found in isolate 3 (resistant) compared to isolate 2 (susceptible).
(XLS)

Table S2 All mutations found in isolate 3 (resistant) compared to isolate 2 (susceptible) with a product quality score of >3,000.
(XLS)
